# Late presenters (LP) in the last 3 years in the anti-AIDS center of Cluj, Romania

**DOI:** 10.1186/1471-2334-14-S4-P7

**Published:** 2014-05-29

**Authors:** Corina Itu, Cristian Jianu, Mirela Flonta, Ligia Ursu, Daniela Marcu

**Affiliations:** 1 Infectious Disease Clinic, Cluj-Napoca, Romania

## 

Objectives. In Europe 50% are late presenters (LP). In a previous study we showed that between 2000-2010 LP represented two thirds of cases and the main transmission route was sexually, with 5% in men who have sex with men (MSM). We proposed to analyze the epidemiologic, clinic and therapeutic issues of late presenters in the last three years and to compare with the previous study. Our center is monitoring about 330 HIV patients.

We performed a retrospective study of the medical records of 113 HIV newly infected adults, admitted in the Infectious Disease Clinic of Cluj between 01.01.2011-30.06.2013. The patients were divided in two samples: sample I as LP and sample II with CD4 cells count more than 350/cmm. Fisher’s exact test was used for statistical analysis: p<0.05 was considered significant.

There were 64 patients (56%) LP and 49 (44%) in sample II. The average age was 37/34 years, sexually transmitted in 56/48, MSM 16/18, (33% MSM in LP), males 43/42, urban area 50/44, symptomatic HIV testing 51/15, life-threatening conditions 24/1, deceased 8/0. We found statistically significant values in the LP for: HIV testing as symptomatic patient (p=0.0001), clinical life-threatening conditions (p=0.0001), rate of deaths (p=0.005); in the sample II we found significant values for HIV testing as screening (p=0.01) and HIV test request (p=0.0009). In the last 3 years there was no significant difference between the two groups: MSM transmission, concordant/discordant couples or celibate, provenience area, age more than 40. No significant values for LP between 2000-2010 and 2011-2013 (p=0.1), but differences in MSM transmission in the last 3 years (p=0.0002).

Between 2000-2013 LP were 56-66% with influence on mortality rate. In the last 3 years the transmission rate on MSM is increasing from 5% to 33%. We have no intravenous drug users. HIV testing with informed consent delays the diagnosis and represents a barrier for earlier HIV testing.

